# Supplementary figures and images for: CRISPR/Cas9 Based Cell-Type Specific Gene Knock-Out in Arabidopsis Roots
Source: Plants (Basel). 2023 Jun 19;12(12):2365. doi: 10.3390/plants12122365 (PMC10303061; doi:10.3390/plants12122365)

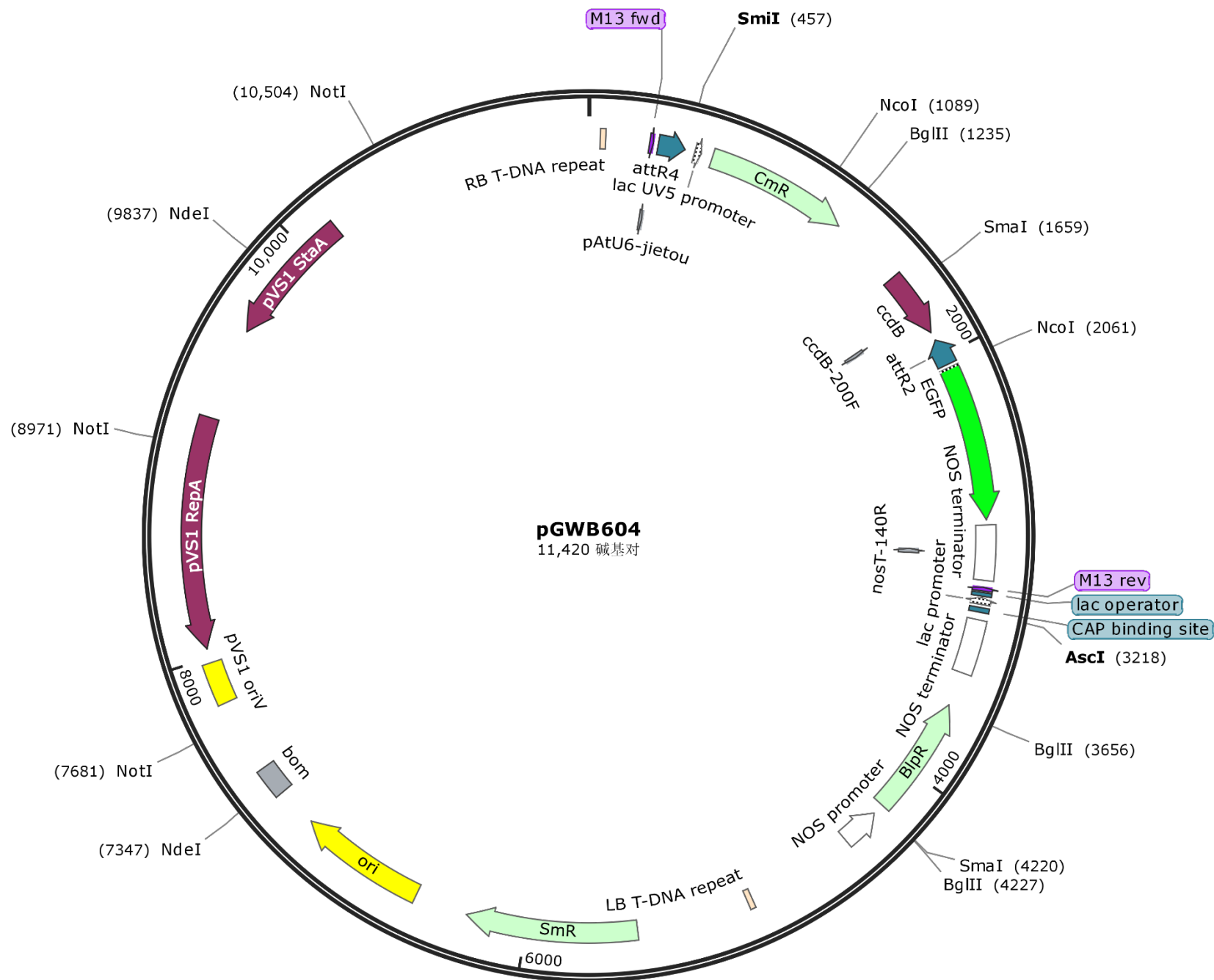

Supplement: Supplementary file 1 [file plants-12-02365-s001.zip › Supplementray files/SI Figure 1.pdf]

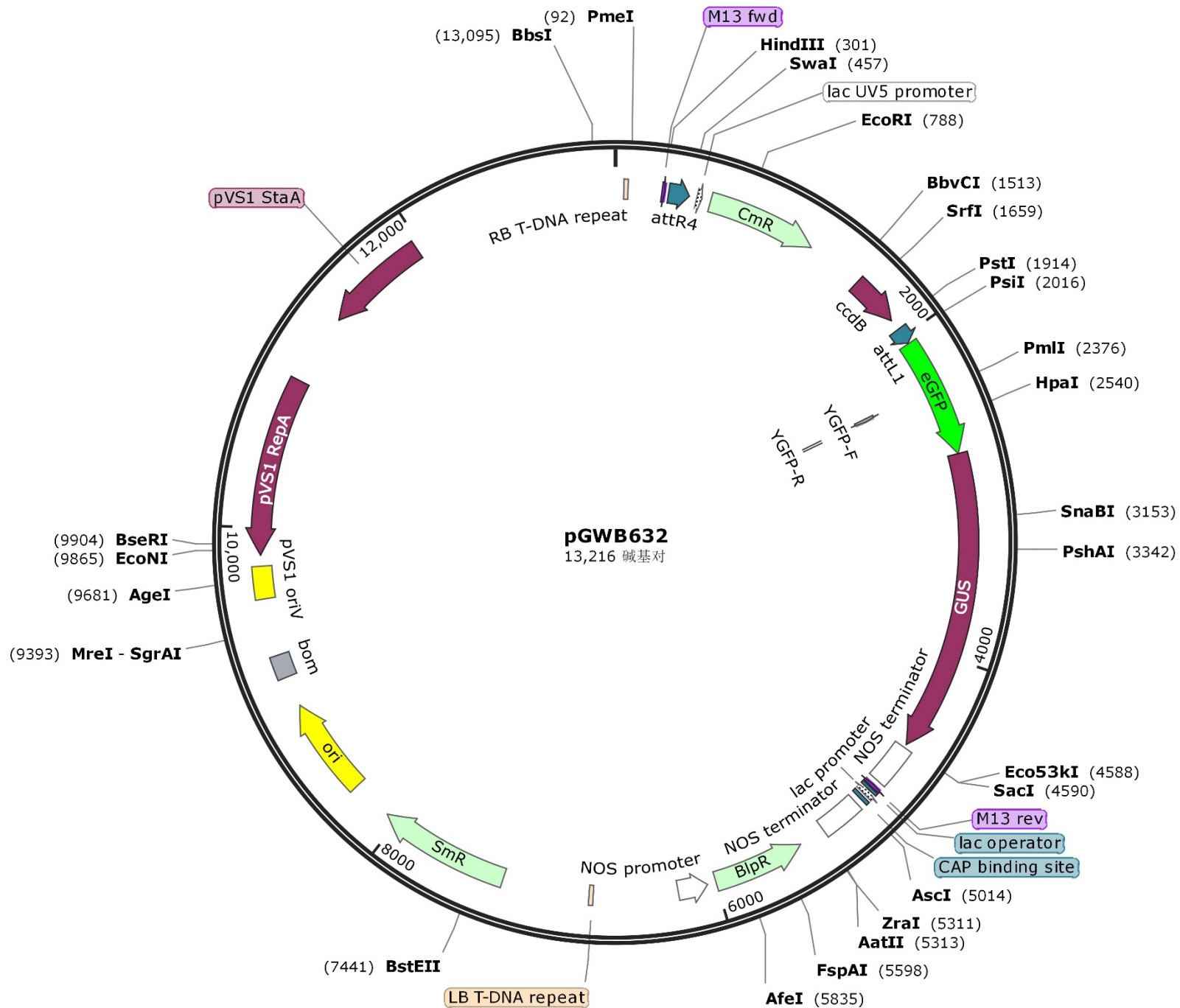

Supplement: Supplementary file 1 [file plants-12-02365-s001.zip › Supplementray files/SI Figure 2.pdf]

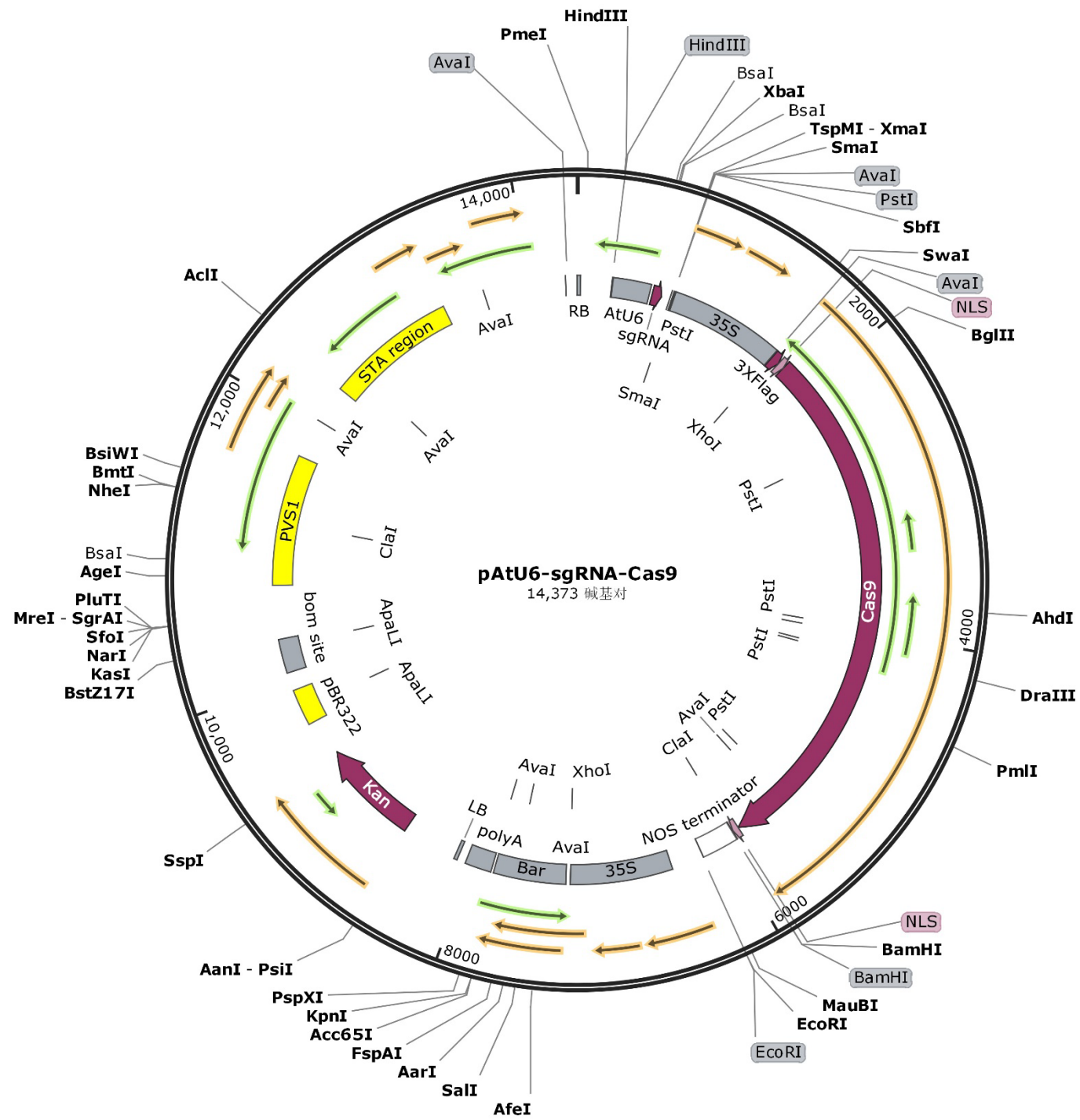

Supplement: Supplementary file 1 [file plants-12-02365-s001.zip › Supplementray files/SI Figure 3.pdf]
